# Short-Term Effect of Inhaled Salbutamol on Heart Rate in Healthy Volunteers

**DOI:** 10.7759/cureus.13672

**Published:** 2021-03-03

**Authors:** Salman A Syed, Nawabzada Zeerak Farhat Sherwani, Bismah Riaz, Javed Iqbal, Manahil Chaudhry, Mohammad Abdullah, Ayesha Malik

**Affiliations:** 1 Department of Medicine, Combined Military Hospital (CMH) Lahore Medical College and Institute of Dentistry, Lahore, PAK; 2 Department of Medicine, Hameed Latif Hospital, Lahore, PAK

**Keywords:** tachycardia, nebulization, salbutamol, short acting beta-agonist

## Abstract

Objective

Tachycardia is a potential side effect of salbutamol inhalation. We aimed to study the short-term effect of salbutamol nebulization on the heart rate of healthy volunteers.

Material and methods

A randomized, single-blinded, placebo-controlled, cross-over study was conducted with 30 healthy volunteers divided into two groups of 15 each. One group was nebulized with salbutamol (2.5 mg/2 ml dilution) while the other group was given normal saline (2 ml). Each arm underwent administration of the opposite drug the following week. Baseline readings of heart rate and blood pressure were taken at zero (T0), seven (T7), 15 (T15), and 30 (T30) minutes.

Results

Thirty volunteers between the ages of 20 and 25 years were studied. The mean heart rate value was higher for nebulized salbutamol at each point as compared to saline. When nebulized with salbutamol, the heart rate had a significant rise (p= < 0.00001) at 15 minutes as compared to saline nebulization.

Conclusion

Salbutamol nebulization, even at a low dose, can lead to a significant increase in heart rate when compared to nebulization with normal saline in healthy individuals.

## Introduction

Salbutamol, a short-acting beta-2-agonist (SABA), is a widely used bronchodilator and is prescribed as a “reliever” medication for asthmatics and chronic obstructive airway disease (COAD) patients [1]. Like every drug, salbutamol can have some systemic effects other than its primary therapeutic effect. One known side effect of this drug is tachycardia.

Salbutamol causes an increase in the heart rate by the following propositioned mechanisms: first, after being absorbed into the bloodstream, it shortens the time of diastole, thus increasing heart rate and, in turn, increasing the myocardial oxygen demand; second, it acts on beta-adrenergic receptors of the heart, resulting in an increased sympathetic outflow [2]. Kallergis and colleagues who studied the electrophysiological changes of the heart in response to salbutamol administration found that the drug can also cause several changes like atrioventricular delay and decreased atrial and ventricular refractoriness [3]. In addition to these cardiovascular effects, an excerpt from the 2018 Global Asthma Report mentioned that changes in medical care, especially the introduction of new asthma medications and inappropriate reliance on such "reliever" medications, may have been a cause of increased or at least fluctuating mortality figures in the last decade [4].

A study done by Waddel et al. proved that uneven ventilation-perfusion ratios are experienced during paroxysms of asthma attacks, which give rise to arterial hypoxia [5]. In order to maintain adequate systemic oxygen perfusion during such acute hypoxic episodes, the heart rate rises whereas the stroke volume remains the same [6]. Burggraaf and colleagues also observed that SABA use in asthmatic patients with hypoxia had an increased likelihood of developing cardiovascular side effects [7]. This highlights that cardiovascular side effects with salbutamol use can be multifactorial. This formed the rationale for conducting a randomized, cross-over study on healthy volunteers. The motivating factor to conduct the study on healthy people was to rule out confounding factors like tobacco use, COAD history, cardiovascular disease, and cardiovascular risk factors on subjects who were under no hypoxic insults, so as to study the direct link between SABA nebulization and heart rate.

## Materials and methods

The study was conducted after due approval from the institute's ethical review committee (ERC# 244/2020) and was conducted in accordance with the principles laid down in the declaration of Helsinki. We conducted a randomized, single-blinded, placebo-controlled, cross-over study in which a total of 30 healthy volunteers having similar daily activities were consecutively recruited after signing informed consent. The study was conducted at a tertiary care hospital with all volunteers aged 20-25 years from January 2 to January 10, 2021. All volunteers had no acute or chronic illnesses, were not on any continuous medication, were non-smokers, and were not using any illicit drugs. Any volunteer enrolled in any exercise training programs, those with a baseline heart rate of more than 100 beats per minute (bpm), and those with a history of allergy to salbutamol were excluded. Female participants were screened based on a detailed gynecological history, and those with the possibility of carrying a pregnancy were also excluded. To ensure randomization, a serial number was allocated to each volunteer, and via a draw method, they were divided into two groups of 15 each, to receive either nebulization with normal saline (2 ml) or salbutamol (2.5 mg/2 ml) for the first session. One week later, a cross-over session was done in the same manner as described ahead.

Before each session, the participants were directed to refrain from any heavy exercise, alcoholic beverages, smoking, and caffeine intake at least 12 hours before the intervention, and abstinence from the above-mentioned factors was confirmed before proceeding by conducting a brief interview. On the day of the session, the participants were taken to a quiet room where they rested for 10 minutes in a sitting position. After they were settled, a brief cardiovascular examination was done, including a recording of the baseline heart rate (by manual count for 60 seconds) and blood pressure (via the auscultatory method at heart level using a mercury sphygmomanometer). One group was given salbutamol (2.5 mg/2 ml dilution) via a face-mask nebulizer with a flow rate of 7.5 liters per minute while the other group was given normal saline (2 ml) via the same instrument and method. The average time of nebulization was about seven minutes. A set of readings of heart rate and blood pressure were taken just before the start of nebulization (T0) and then at seven minutes (T7), 15 minutes (T15), and 30 minutes (T30).

If at any point, the heart rate exceeded the age-adjusted limit (220 beats per minute - age) or any pulse irregularity was noted, the nebulization was discontinued. Each volunteer was also observed for any side effects, including palpitations, tremors, headache, nausea, vomiting, or any untoward symptom. This formed the secondary endpoint of the study. Descriptive and analytic statistics were drawn via the Social Package for the Social Sciences (SPSS) version 25 (IBM Corp., Armonk, NY). The T-test was used to determine any significant difference in heart rate variation.

## Results

**Figure 1:**
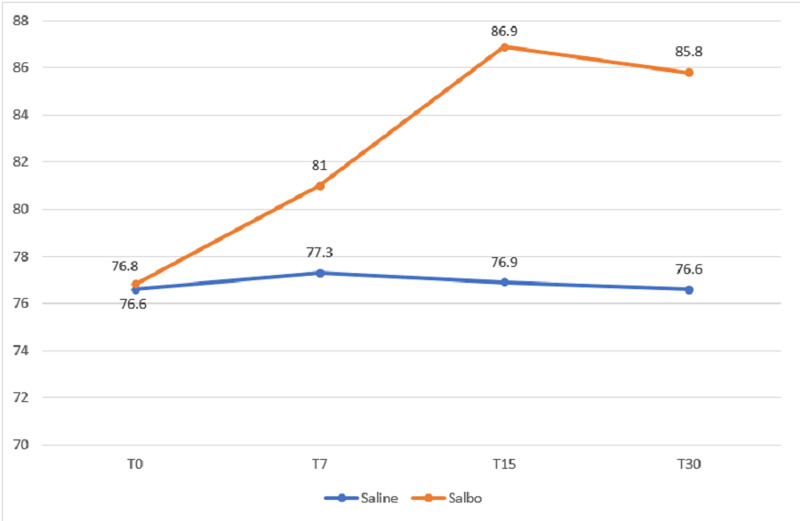
Changes in heart rate following nebulization with salbutamol and saline

This single-blinded, randomized controlled study was conducted on healthy volunteers between the ages of 20 and 25 years. The male to female ratio was 1:1 and the mean body mass index (BMI) was 23.6. Heart rate (HR) and blood pressure (BP) were recorded at T0, T7, T15, and T30 (Table 1). Heart rate variability was observed in volunteers receiving salbutamol, and a steep rise in the mean HR of 10 bpm was seen at 15 minutes (p<0.00001), and this was sustained at T30 (Figure 1). In the saline group, a very slight rise (p=0.130) was seen from T0 (74.85 bpm) to T7 (76.0 bpm), which fell back to near baseline at T30 (Figure 1).

**Table 1:** Changes in heart rate and blood pressure following the administration of salbutamol and normal saline (Mean ± SD) T0 – readings at baseline, T7 – readings at seven minutes, T15 – readings at 15 minutes, T30 – readings at 30 minutes, bpm – beats per minute, mmHg – millimeters of Mercury

		T0	T7	T15	T30
Pulse (bpm)	Salbutamol	76.8 ± 7.2	81.0 ± 8.0	86.9 ± 10.2	85.8 ± 9.6
	Saline	76.6 ± 6.1	77.3 ± 5.7	76.9 ± 5.5	76.6 ± 5.7
Systolic blood pressure (mmHg)	Salbutamol	112.2 ± 12.8	113.2 ± 11.6	112.2 ± 11.1	111.9 ± 10.1
	Saline	112.3 ± 12.1	113.3 ± 11.3	111.5 ± 10.9	110.8 ± 10.3
Diastolic blood pressure (mmHg)	Salbutamol	75.0 ± 7.4	76.2 ± 7.6	72.7 ± 9.6	74.8 ± 9.8
	Saline	75.8 ± 8.5	74.2 ± 9.1	73.5 ± 9.0	74.0 ± 9.2

A change in heart rate and blood pressure after drug/placebo administration was determined. The maximum change was noted at T15 and, hence, this was kept as a benchmark for comparison with the baseline readings (Table 2). We observed a rise in heart rate following salbutamol administration, as mentioned previously. However, in both the groups, there was a fall in diastolic blood pressure, but considering this was seen in both the groups and none of the volunteers exhibited any symptoms, it seemed insignificant. None of the participant's heart rate exceeded the age-adjusted limit during testing. No untoward side effects (such as flushing, palpitations, or dizziness) or drug reactions were observed in the groups while receiving salbutamol.

**Table 2:** Mean change in heart rate and blood pressure following nebulization with salbutamol and normal saline T0 – readings at baseline, T7 – readings at 7 minutes, T15 – readings at 15 minutes, T30 – readings at 30 minutes, bpm – beats per minute, mmHg – millimeters of Mercury

		T0	T15	∆
Mean heart rate (bpm)	Salbutamol	76.8	86.9	10.1
	Saline	76.6	76.9	0.3
Mean systolic blood pressure (mmHg)	Salbutamol	112.2	112.2	0
	Saline	112.3	111.5	-0.8
Mean diastolic blood pressure (mmHg)	Salbutamol	75.0	72.7	-2.3
	Saline	75.8	73.5	-2.3

## Discussion

Salbutamol is a beta-2 agonist and is widely used as a bronchodilator in the treatment of patients with bronchial asthma and COAD. We aimed to analyze the acute effects of salbutamol inhalation on HR and BP in healthy volunteers. Our results show that salbutamol nebulization leads to a significant increase in HR as compared to placebo. The dose used in our study was lesser than the commonly used clinical dose (5 mg). This indicates that tachycardia is a significant feature even at lower doses.

Our results are in coherence with those found by Cekici et al. [2], Jartti et al. [8], Edgel et al. [9], who also noted an increase in heart rate following salbutamol inhalation. The aforementioned studies used transthoracic impedance cardiography, determining pulse wave velocity, and heart rate variability (HRV) analysis to measure the hemodynamic parameters. Another difference between these studies and ours was that we used a face mask for delivering the drug to make the study resemble actual acute clinical settings while Cekici et al. [2] and Jartti et al. [8] used metered-dose inhalers (MDI). Kaya et al. [10] found no significant rise in heart rate following salbutamol administration.

There were certain limitations to our study. Beta-2 agonists lead to decreased potassium levels, which can cause electrophysiological changes that may affect cardiovascular parameters [11]. The measurement of serum potassium levels was not part of this study’s framework. Moreover, because the focus was on immediate effects, heart rate recovery was not studied at length so a valid estimate of the time the drug affected the system could not be inferred. Nevertheless, we used a crossover sampling technique, something which hasn’t been done previously, to avoid intervention bias.

## Conclusions

Salbutamol nebulization, even at a low dose, can lead to a significant increase in HR when compared to nebulization with normal saline in healthy individuals. The short-term cardiovascular side effects of SABA use, especially tachycardia, become important in clinical settings where it can potentiate more adverse complications.
